# Investigating the link between trait procrastination and college students’ sleep quality: the mediating role of self-efficacy and executive function

**DOI:** 10.3389/fpsyt.2025.1653871

**Published:** 2026-01-08

**Authors:** Yan Wang, You Chong, Ruixue Men, Fei Li, Hongbing Pan, Xuan Xu

**Affiliations:** 1 College of Traditional Chinese Medicine, Changchun University of Chinese Medicine, Changchun, Jilin, China; 2 School of Marxism, Changchun University of Chinese Medicine, Changchun, Jilin, China; 3 Department of Criminal Science and Technology, Jilin Police College, Changchun, Jilin, China; 4 School of Marxism, Changchun University of Architecture and Engineering, Changchun, Jilin, China

**Keywords:** college students, trait procrastination, self-efficacy, executive function, sleep quality

## Abstract

**Purpose:**

As a personality trait characterized by an irrational tendency to avoid, trait procrastinators usually have poor time management. When they face the dilemma of stopping to sleep or working inefficiently under the threat of deadlines, they usually make difficult choices, leading to poor sleep. The purpose of this study is to explore the comprehensive effects of trait procrastination, self-efficacy and executive function on the sleep quality of college students, and to investigate the mediating role of self-efficacy and executive function.

**Material and methods:**

745 college students were surveyed using the short version of the General Procrastination Scale, General Self-Efficacy Scale, Chinese version of Geurten Executive Function Questionnaire and the Pittsburgh Sleep Quality Index. Descriptive statistics, correlations analysis and multicollinearity diagnosis were conducted. To test the proposed hypotheses, a chain mediation model was built, and a bootstrap analysis was conducted using Process 4.2 in the SPSS macro program.

**Results:**

1) Trait procrastination significantly negatively predicted sleep quality. 2) Self-efficacy mediated the relationship between trait procrastination and sleep quality. 3) Executive function mediated the relationship between trait procrastination and sleep quality. 4) Self-efficacy and executive function played a chain mediation role between trait procrastination and sleep quality. 5) When self-efficacy acted as the sole mediating factor, there was a masking effect.

**Conclusion:**

A higher level of trait procrastination was associated with lower self-efficacy, impaired executive function, and poorer sleep quality, suggesting that trait procrastination adversely affects not only mental health but also higher-order cognitive and physiological functions. Self-efficacy weakened or masked the direct relationship between trait procrastination and sleep quality. These findings offer valuable insights into the dual protective role of self-efficacy against trait procrastination affecting sleep quality. Interventions aimed at improving executive function have great potential to enhance the sleep quality of college students with procrastination problems.

## Introduction

In the digital era, the pervasive consumption of short-form videos on smartphones provides immediate information and emotional stimulation. However, this fragmented mode of information engagement diminishes sustained attention (1–3), impairs deep thinking (4), and weakens goal-directed behavior (5), while easy access to emotional stimuli reduces the capacity for delayed gratification (6). Consequently, individuals prone to short-form video addiction often struggle to complete tasks requiring prolonged concentration, such as academic assignments (7, 8). In China, college students are among the most affected by this trend. Meanwhile, within highly competitive academic environments, college students must consistently develop plans and meet deadlines for coursework, internships, and examinations, thereby facing elevated risks of failure and negative evaluation (9). Under the dual pressures of digital distraction and academic demands, many students find it difficult to disengage from their devices and frequently delay preparatory work until the last minute (10, 11).

Procrastination refers to the individual voluntarily delaying the start or completion of planned tasks due to the anticipation of unfavorable consequences (12), characterized by an irrational tendency to avoid (13). It manifests in two common forms: active and passive procrastination. Active procrastination involves a deliberate choice to delay tasks to harness heightened motivation under pressure, representing a strategic and adaptive approach (14, 15). In contrast, passive procrastination is marked by an unwillingness to delay yet an inability to act promptly, often accompanied by negative emotions such as guilt and shame (16, 17). While appropriate procrastination may serve as a manageable strategy, chronic and excessive delay can evolve into trait procrastination, which is one of the predictive factors for subclinical psychiatric symptomatology (18–20).

Trait procrastination has been commonly viewed as a type of personality trait (personality tendency), characterized by sensitive to instant rewards (21), self-doubt (22), and an excessive fear of failure (23, 24). Its origins may be traced to early parenting styles (25–27). It reflects a persistent pattern of unnecessarily postponing tasks and goals (28–30), marked by avoidance, indecision, and inaction, which can adversely affect academic, occupational, and social functioning. In some extreme cases, the behavior results in consequences associated with other more traditional personality disorders such as low conscientiousness (31–33), high impulsivity (34, 35), high neuroticism (36), exorbitant perfectionistic concerns (37, 38).

Research indicates that problematic procrastination is highly prevalent and poses serious risks to physical and mental health (39–41), which has progressively developed into a matter of global concern (42). The prevalence of problematic procrastination behavior appears to be high in China, especially among young adults. One study reported that over 75% of Chinese university students aged over 18 years old (N = 819) admitted to engaging in academic procrastination (43). Trait procrastination has been associated with numerous negative outcomes, such as depression (44) and anxiety (13), burnout (45, 46), and poor sleep quality. Given that sleep quality is a critical determinant of overall health and well-being, it is important to understand the mechanisms linking procrastination to sleep disturbances. To date, few studies have explored these pathways. One investigation highlighted the role of rumination and depressive mood in this relationship (47), but no research has yet considered the joint influence of positive psychological variables (e.g., self-efficacy) and higher-order cognitive functions (e.g., executive function). Therefore, this study examines the effect of trait procrastination on sleep quality among Chinese youth, and investigates the potential mediating roles of self-efficacy and executive function.

Sleep is a natural, physiologically regulated process. From a cognitive neuroscience perspective, it depends on homeostatic synaptic regulation in the prefrontal cortex, which is related to self-control, rewards, and emotions. Good sleep quality helps to consolidate adaptive cognitive, behavioral and emotional patterns in daily life. However, individuals with high levels of procrastination may exhibit altered neural connectivity and impaired prefrontal functioning (48), including deficits in top-down control. Indeed, sleep dysfunction is increasingly documented among those who procrastinate habitually (49, 50).

Empirical studies have consistently linked procrastination with poorer sleep outcomes. For example, a large cross-sectional study of U.S. adolescents and young adults (N = 8742) found that higher procrastination was associated with worse sleep quality (50). Another study reported that procrastination was positively associated with both short video addiction and sleep quality (as measured by the PSQI) (51). Procrastination has also been linked to poor sleep hygiene (52). Thus, the present study seeks to confirm the association between trait procrastination and sleep quality, and to extend current knowledge by examining indirect effects via self-efficacy and executive function.

Self-efficacy reflects an individual’s confidence in their ability to successfully execute behaviors necessary to achieve desired outcomes. Low self-efficacy is associated with negative consequences among young adults, including trait procrastination. Multiple studies have reported significant negative correlations between academic procrastination and academic self-efficacy (53–55). Furthermore, the relationship between self-efficacy and sleep quality has also been reported in previous research. For example, one research proved that higher self-efficacy and problem-focused coping strategies could positively predicted better sleep quality (56). Another study revealed that decreasing sleep quality was associated with low academic self-efficacy (r=-.121, p<0.01) (57).

Executive function encompasses a set of advanced cognitive abilities/processes related to the frontal lobe of the brain, including planning, organization, problem-solving, working memory, and decision-making, etc. These processes allow individuals to optimize their behavioral performance in new ways in the daily environment. Recent studies indicated that procrastination is associated with deficits in multiple executive domains, such as working memory, organization, time management, emotional control, task initiation, sustained attention and cognitive flexibility (58, 59). Two studies demonstrated that both active and passive procrastinators show impairments in inhibitory control and other executive abilities (60, 61). Behavioral genetic studies further suggested that procrastination and poor executive functioning share common genetic underpinnings (62). In terms of sleep, a scoping review found cognitive decline might contribute to the onset of insomnia, further deteriorating sleep quality (63). For instance, poor cognitive control has been linked to sleep quality via trait mindfulness and emotional stress (64), and lower executive functions were associated with increased pre-sleep negative cognitions, which in turn predicted more sleep problems (65).

Previous research has reported that both self-efficacy and executive function are related to trait procrastination behavior. Moreover, self-efficacy was also associated with executive function. For example, higher self-efficacy has been shown to positively influence executive function (66), and self-efficacy longitudinally predicts perceived cognitive impairment in both general cognitive function and executive function (67). However, no prior study has explored the sequential mediating role of self-efficacy and executive function in the relationship between trait procrastination and sleep quality.

### Theoretical background

The Time Decision Model (TDM) of procrastination offers a theoretical framework for understanding the link between procrastination and sleep quality (68, 69). According to TDM, procrastination arises from a motivational conflict between the impetus to act and the impetus to delay. This competition can be understood as a trade-off between the anticipated utility of a task’s positive outcome and the immediate aversiveness of the task process itself (70). A core function of procrastination is to actively postpone engagement with aversive tasks process (71). Sleep, as an essential physiological cycle for energy restoration, presents a critical decision point for individuals with high levels of trait procrastination. They face a recurring dilemma: either cease working to sleep and regain energy, or sacrifice sleep to continue working. Under the time deadline threats, they were torn between difficult choices, which ultimately led to poor sleep (72–74).

From the perspective of an extended procrastination-health model, trait procrastinators accumulate negative experiences from repeated coping failures, which undermines their confidence in task completion. Trait procrastination fosters feelings of frustration and powerlessness, reinforcing self-doubt and negative self-evaluation (75), thereby resulting in low self-efficacy (76). This diminished self-efficacy reinforces a pattern of avoidance and retreat. Previous work suggested that procrastination is associated with weak health intentions, mediated by lower health-specific self-efficacy (77). Trait procrastination was associated with reduced motivation for health behavior change and poorer outcome expectancies regarding health-related goals (78). Consequently, individuals high in trait procrastination exhibit lower motivation for health-sustaining behaviors, making them more susceptible to delaying sleep or experiencing sleep-related difficulties.

Executive function comprises higher-order cognitive abilities that enable individuals to regulate thoughts, actions, and emotions toward goal-directed behavior (79). Trait procrastination is increasingly conceptualized as a failure of self-regulation and volition (80), characterized by maladaptive coping (81), poor self-control (82) impaired attentional control (83, 84), goal management failures (62) and an avoidance goal orientation (85), which shares common components with executive functions. According to the Synergistic and Differentiation Theory of Executive Function, optimal performance arises from the integrated collaboration of various cognitive sub-functions; when these components fail to work synergistically, it leads to internal conflict and impedes goal achievement (86). In the context of trait procrastination, diminished self-efficacy may disrupt the synergistic operation of executive processes. For instance, in making decisions about sleep, the executive system may become dysregulated, unable to effectively coordinate the competing demands of task completion and physiological need, thereby compromising healthy sleep behavior.

### Hypotheses development

**Figure 1:**
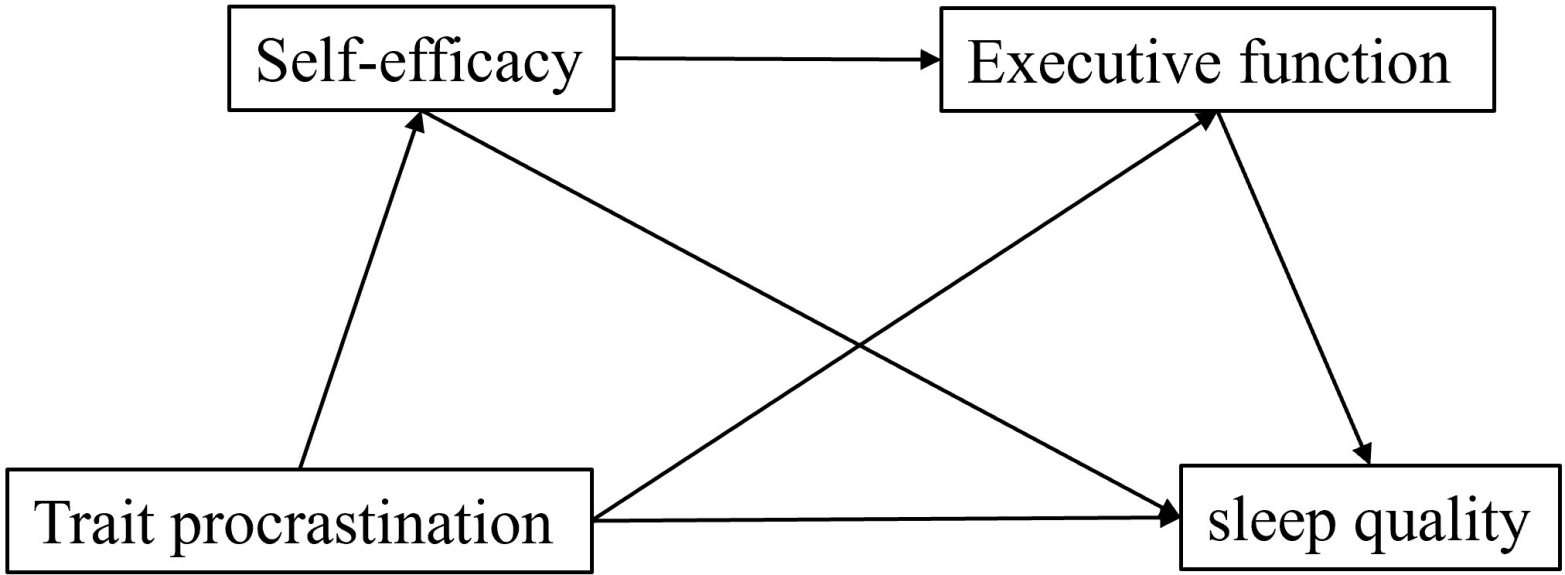
The Hypothesized chain mediation model.

Based on the theoretical framework and literature review presented above, this study aims to address the identified research gap by empirically examining the impact of trait procrastination on the sleep quality of Chinese young adults, and by testing the potential chain mediating roles of self-efficacy and executive function. A conceptual chain mediation model was constructed to guide this investigation, as illustrated in **Figure 1**. The following hypotheses were proposed and tested:

Hypothesis 1: Trait procrastination will be negatively associated with sleep quality.

Hypothesis 2: Self-efficacy will be positively associated with sleep quality.

Hypothesis 3: Self-efficacy will mediate the relationship between trait procrastination and sleep quality.

Hypothesis 4: Executive function will be positively related to sleep quality.

Hypothesis 5: Executive function will mediate the relationship between trait procrastination and sleep quality.

Hypothesis 6: Self-efficacy and executive function will sequentially mediate the association between trait procrastination and sleep quality.

## Material and methods

### Participants

This cross-sectional survey was conducted during November 2024, utilizing a convenience sampling method. Participants were recruited from three public universities in Changchun City, Jilin Province, China. Undergraduate students across different majors and grade levels were invited to participate in the study. The inclusion criteria for the research participants were: (1) full-time undergraduate students; (2) voluntary willingness to participate; (3) no previous history of mental illness. The exclusion criteria included: (1) a history of major diseases; (2) Have taken or are taking psychotropic drugs in the past 2 months; (3) a background in psychology education. The exclusion of psychology majors is a common practice to minimize potential biases in self-report measures, such as experimenter expectations effects, which may arise from participants’ prior knowledge of psychological assessment, thereby safeguarding the validity of the results.

A total of 812 undergraduate students voluntarily completed the survey. After data cleaning, 745 valid questionnaires were retained, resulting in an effective response rate of 91.7%. Moreover, the subject-to-item ratio reached 10:1, substantially exceeding the commonly recommended minimum threshold of 5:1 (87–89). Therefore, the standards that the sample size should meet have been fully met, thereby ensuring the reliability of the study results.

Demographic characteristics of the sample are summarized in **Table 1**. The sample included 351 female (47.1%) and 394 male (52.9%) participants. In terms of age distribution, the largest group consisted of 18-years-old (n = 207, 27.8%), followed by 19-year-olds (n = 361, 48.5%). Regarding academic standing, freshmen (n = 238, 31.9%) and sophomores (n = 366, 49.1%) constituted the majority. A total of 470 participants (63.1%) reported urban residency, while 275 (36.9%) were from rural areas. In addition, 367 participants (49.3%) were only children, and 378 (50.7%) had siblings.

**Table 1:** Demographic information of effective subjects.

Variables	Specific category	Sample size	Percentage
Gender	Female	351	47.1%
	Male	394	52.9%
Age	<18 years	27	3.6%
	18 years	207	27.8%
	19 years	361	48.5%
	20 years	112	15.0%
	>20 years	38	5.1%
Grade	Freshman	238	31.9%
	Sophomore	366	49.1%
	Junior	116	15.6%
	Senior	25	3.4%
Place of Residence	Rural	275	36.9%
	Urban	470	63.1%
Only Child Status	Only child	367	49.3%
	Not an only child	378	50.7%

### Procedure

The data collection procedure was conducted as follows. At first, the principal investigator received standardized training on survey administration to ensure consistency in methodology, key procedures, and timing. Next, at the beginning of the formal survey, the investigator in charge provided a unified introduction to all participants, explaining the research purpose, significance, and scope of application. Participants were informed of their rights, including the right to withdraw at any time without penalty. Then, Data collection was organized by class units using Wenjuanxing (www.wjx.cn), a widely used online survey platform in China. Participants accessed the survey via a shared link and completed the questionnaire voluntarily without compensation. Anonymity was ensured to protect participant privacy. Finally, upon collection of the questionnaires, quality assurance was performed in adherence to predefined screening principles, including the verification of reverse-scored items, identification of patterned responses, and elimination of incomplete surveys.

### Measures

#### Trait procrastination

Trait procrastination was measured using the Short General Procrastination Scale (SGPS) (90), which consists of nine items. Participants respond these items (eg, “When I finish tasks with deadlines approaching, I often waste time doing other things”) on a five-point scale ranging from 1 (completely not matched) to 5 (absolutely matched). The total score was calculated, with higher scores indicating a greater tendency toward trait procrastination. In the present study, the SGPS demonstrated good internal consistency (Cronbach’s α = 0.75).

#### Self-efficacy

Self-efficacy was assessed using the 10-item General Self-Efficacy Scale(GSE) (91). All items (eg, “If I try my best, I can always solve the problem”) were rated on a four-point Likert scale ranging from 1 (Not at all true) to 4 (Exactly true). Higher total scores indicate stronger general self-efficacy. The scale showed excellent internal consistency in this study (α = 0.95).

#### Executive function

Executive function was evaluated using the Geurten—Questionnaire of Executive Functioning in Chinese College Students(G—QEF—C) (92), a version adapted by Professor Xue and colleagues in 2022 from the original Geurten Executive Function Questionnaire (93). The modified scale contains 35 items (after removing the 22nd item of the original version) and assesses eight dimensions: emotional regulation, impulse control, attention focus, self-monitoring, theory of mind, planning initiation, cognitive flexibility, and working memory. Exploratory factor analysis (EFA) indicated that the eight factors had eigenvalues greater than 1, collectively explaining 52.80% of the variance. Confirmatory factor analysis (CFA) supported the eight-factor model (χ²/df = 1.93, RMSEA = 0.04, CFI = 0.92), which provided a better fit than a single-factor model (χ²/df = 8.62, RMSEA = 0.11, CFI = 0.36). Furthermore, the scale also exhibited significant positive correlations with the Behavior Rating Inventory of Executive Function-Adult Version (r=0.77) and the Barratt Impulsiveness Scale (r=0.71). Each item (eg, “I found it difficult for me to be wholehearted while studying”) was rated on a four-point scale ranging from 1 (never) to 4(Almost always). Reverse scoring was applied in this study, and higher total scores indicate better executive function. The internal consistency reliability of the G-QEF-C was very good in this sample (α=0.92).

#### Sleep quality

The Pittsburgh Sleep Quality Index (PSQI) (94) were used to assess sleep quality. The PSQI contains 18 items grouped into seven components: subjective sleep quality, sleep latency, sleep duration, sleep efficiency, sleep disturbances, use of sleep medication, and daytime dysfunction (94). Respondents reported their sleep experiences over the past month (eg, “Wake up easily at night or early”) on a four-point scale ranging from 0 (never) to 3 (above 3 times per week). The total score of this scale was calculated with higher scores indicating poorer sleep quality. The PSQI showed good internal consistency in the current study (α=0.72).

### Data analysis

Data were analyzed using SPSS 27.0 and Mplus 8.3. Initially, common method bias was assessed via Harman’s single-factor test in SPSS and a single-factor CFA in Mplus 8.3. Subsequently, descriptive statistics and Pearson correlations were conducted to examine the relationships among trait procrastination, self-efficacy, executive function and sleep quality. And multicollinearity among predictor variables was evaluated using the variance inflation factor (VIF). Ultimately, a chain mediation model was tested using Model 6 of the PROCESS macro (version 4.2) for SPSS (95), with trait procrastination as the independent variable, self-efficacy and executive function as the mediators, and sleep quality as the dependent variable. Regression coefficients were tested using bias-corrected bootstrap confidence intervals based on repeating sampling 5000 times. A two-sided significance level of α = 0.05 was adopted. All variables were standardized before analysis.

## Results

### Common method biases

Common method bias is a known concern in self-reported data and was thus evaluated using Harman’s single-factor test (96). The results revealed ten factors with eigenvalues greater than 1, with the largest factor accounting for 21.51% of the variance, which was well below the critical threshold of 50% (97, 98). To further validate these findings, a single-factor CFA was conducted, which indicated poor model fit (RMSEA = 0.175>0.08, CFI = 0.252<0.9, TLI = 0.204<0.9, SRMR = 0.238>0.08, X^2^/df=23.85). These results collectively suggest that common method bias was not a serious concern in the present dataset. The high reliability of the research results was ensured.

### Preliminary analyses

Kline proposed a condition that is considered to be approximately normally distributed, which includes the absolute value of kurtosis not exceeding 10 and the absolute value of skewness not exceeding 3 (99). In this study, both skewness and kurtosis for normality test were used. The results display that absolute values of two measures are below 2 and within broadly acceptable statistical ranges (98–101). Therefore, the data were approximately normally distributed and thus suitable for subsequent parametric analyses.

Descriptive statistics (ie, means and standard deviations), and Pearson’s bivariate correlational coefficients for all observed variables are presented in **Table 2**. For demographic variables, age was significantly correlated with executive function (*r* = -0.115, *p* < 0.01), with older reporting lower executive function than younger. Trait procrastination was positively correlated with poor sleep quality (r=0.311, p<0.01) and negatively correlated with self-efficacy (*r* = -0.237, *p* < 0.01) and executive function (*r* = -0.615, *p* < 0.01). Executive function was negatively correlated with poor sleep quality (*r* = -0.358, *p* < 0.01). Furthermore, self-efficacy was significantly positively correlated with executive function (*r* = 0.271, *p* < 0.01), but not significantly correlated with sleep quality (*p_s_* > 0.05).

**Table 2:** Descriptive statistics and intercorrelations among variables.

Variables	M	SD	1	2	3	4
1 Age	18.95	1.016	-			
2 Trait procrastination	21.64	6.211	0.063	-		
3 Self-efficacy	27.34	6.592	-0.021	-0.237^**^	-	
4 Executive function	70.50	12.375	-0.115^**^	-0.615^**^	0.271^**^	-
5 Sleep quality	4.38	3.140	-0.041	0.311^**^	-0.020	-0.358^**^

**P < 0.01; Sleep quality: Higher scores indicate poorer sleep quality.

When multicollinearity exists, the mediation effect may be affected, thereby impacting the accuracy and reliability of the mediation model. Since the VIF is unrelated to the distribution pattern of the outcome variable, it was employed to detect multicollinearity among predictor variables in this study. The results of three linear regression models (**Table 3**) showed that all VIF values of predictor variables were below the conventional threshold of 5 (the maximum VIF = 1.647), Therefore, there were no severe collinearity problems among the independent variables in the three models.

**Table 3:** Multicollinearity diagnosis results.

Outcome	Predictors	Unstandardised coefficients		Standardised coefficient	t	*p*	Multicollinearity diagnosis	
		β	Standard error	β			Tolerance	VIF
SE	TP	-0.251	0.038	-0.237	-6.640	<0.001	1.000	1.000
R=0.237		R^2^ = 0.056		Adjusted R^2^ = 0.055			F=44.093	
EF	TP	-1.164	0.059	-0.584	-19.881	<0.001	0.944	1.059
	SE	0.248	0.055	0.132	4.503	<0.001	0.944	1.059
R=0.629		R^2^ = 0.395		Adjusted R^2^ = 0.394			F=242.538	
SQ	TP	0.081	0.022	0.161	3.754	<0.001	0.619	1.615
	SE	0.043	0.017	0.090	2.548	<0.05	0.917	1.091
	EF	-0.078	0.011	-0.305	-7.062	<0.001	0.607	1.647
R=0.406		R^2^ = 0.165		Adjusted R^2^ = 0.162			F=48.554	

Sleep quality: Higher scores indicate poorer sleep quality. TP, Trait procrastination; SE, Self-efficacy; EF, Executive function; SQ, Sleep quality.

### The mediation model analysis

**Figure 2:**
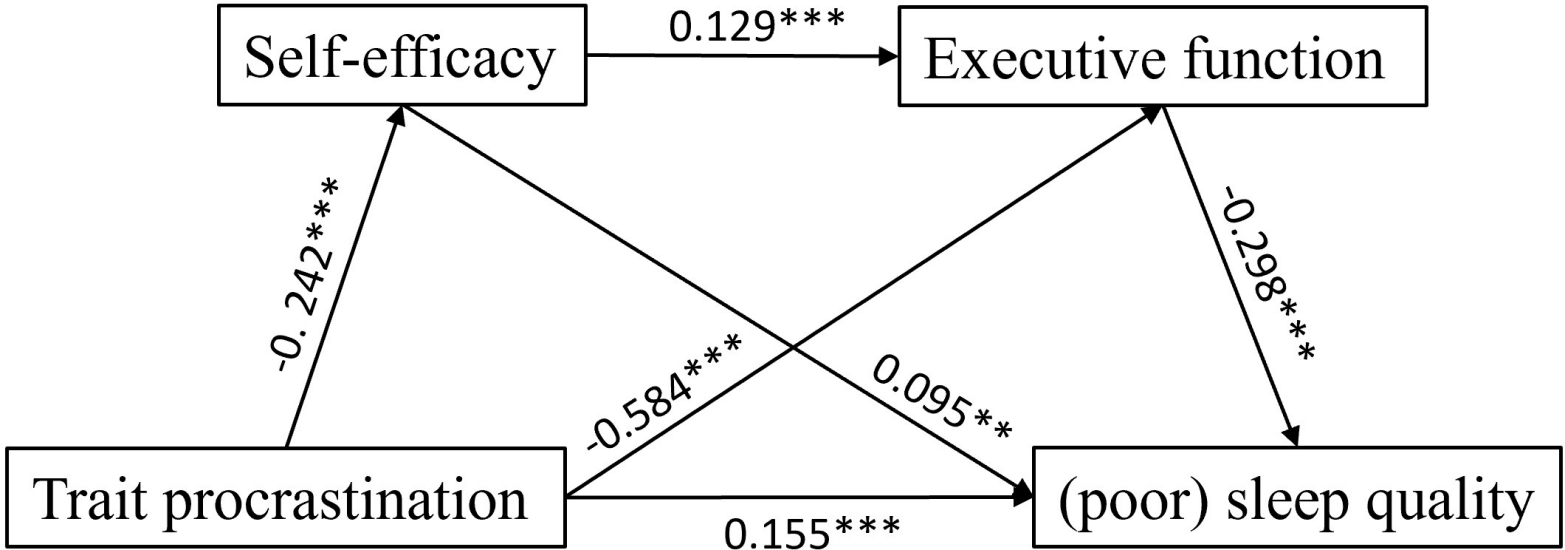
The chain mediation model. All estimated parameters were standardized; Sleep quality: Higher scores indicate poorer sleep quality; ***P < 0.001, **P < 0.01.

The chain mediation model was tested using PROCESS Macro Model 6, controlling for age and gender. The results are summarized in **Figure 2** and **Table 4**. Trait procrastination had a significant total effect on poor sleep quality (β = 0.315, p < 0.001), supporting Hypothesis 1. It also negatively predicted self-efficacy (β = –0.242, p < 0.001) and executive function (β = –0.584, p < 0.001). Executive function negatively predicted poor sleep quality (β = –0.298, p < 0.001), supporting Hypothesis 4. Self-efficacy positively predicted executive function (β = 0.129, p < 0.001). Contrary to Hypothesis 2, self-efficacy positively predicted poor sleep quality (β = 0.095, p < 0.01). After controlling for procrastination, self-efficacy, and gender, age demonstrated a significant negative association with executive function (β = –0.070, p < 0.05). This suggests that, among college students with comparable levels of procrastination, self-efficacy, and gender, increased age is associated with lower executive function. Furthermore, after accounting for procrastination, self-efficacy, executive function, and gender, age remained a significant negative predictor of poor sleep quality (β = –0.081, p < 0.05). This result indicates that older students tended to report better sleep quality relative to their younger counterparts, when other modeled factors were held constant.

**Table 4:** Analysis of the chain mediation model.

Regression equation		Fitness index			Significance of regression coefficients			
Outcome variables	Predictor variables	R	R^2^	F	β	SE	LLCI	ULCI
Sleep quality	Gender	0.317	0.100	27.537	-0.001	0.070	-0.140	0.137
	Age				-0.060	0.035	-0.128	0.008
	Trait procrastination				0.315***	0.035	0.246	0.384
Self-efficacy	Gender	0.244	0.060	15.650	0.120	0.072	-0.021	0.261
	Age				0.001	0.035	-0.069	0.070
	Trait procrastination				-0.242***	0.036	-0.312	-0.171
Executive function	Gender	0.635	0.403	124.650	0.079	0.058	-0.034	0.192
	Age				-0.070*	0.028	-0.126	-0.015
	Trait procrastination				-0.584***	0.029	-0.641	-0.526
	Self-efficacy				0.129***	0.029	0.072	0.187
Sleep quality	Gender	0.396	0.157	27.414	0.015	0.069	-0.119	0.150
	Age				-0.081*	0.034	-0.147	-0.015
	Trait procrastination				0.155***	0.043	0.069	0.240
	Self-efficacy				0.095**	0.035	0.026	0.165
	Executive function				-0.298***	0.044	-0.384	-0.212

All estimated parameters were standardized; Sleep quality: Higher scores indicate poorer sleep quality; *P < 0.05, **P < 0.01, ***P<0.001.

### Mediation effect test

Direct, indirect, and total effects are shown in **Table 5**. The direct effect of trait procrastination on sleep quality was statistically significant (0.155, 95% CI [0.069, 0.240]). The indirect effects of trait procrastination on sleep quality via self-efficacy alone (-0.023, 95% CI [-0.042, -0.004]), executive function alone (0.174, 95% CI [0.118, 0.233]) and both self-efficacy and executive function (0.009, 95% CI [0.004, 0.016]), respectively, were also found significant, which supported H1, H3, H5 and H6.

**Table 5:** Decomposition and estimates of total, direct, and indirect effects.

Impact paths	Standardized effect	BootSE	95%CI		Proportion of total effect (%)
			LLCI	ULCI	
Direct effect	0.155	0.043	0.069	0.240	49.08
TP→SE→SQ	-0.023	0.010	-0.042	-0.004	7.31
TP→EF→SQ	0.174	0.030	0.118	0.233	55.27
TP→SE→EF→SQ	0.009	0.003	0.004	0.016	2.95
Total indirect effect	0.160	0.031	0.102	0.224	50.92
Total effect	0.315	0.035	0.246	0.384	100.00

Sleep quality: Higher scores indicate poorer sleep quality.

TP, Trait procrastination; SE, Self-efficacy; EF, Executive function; SQ, Sleep quality.

Furthermore, the direct effect of trait procrastination on sleep quality accounted for 49.08% of the total effect. The mediating effects −0.023 (TP→SE→SQ), 0.174 (TP→EF→SQ) and 0.009(TP→SE→EF→SQ) accounted for 7.31% and 55.27%, 2.95% of the total effect (0.315), respectively. According to the step diagram proposed by Zhao et al. (102), the effect value of the indirect impact path (TP→SE→SQ) is opposite to the total effect and the direct effect values, indicating a competitive mediation (−0.023, 7.31%); whereas the other two indirect impact paths showed complementary mediation (0.183, 58.23%). It’s worth noting that the competitive effect is also known as the masking effect. The masking effect refers to the situation where, after the introduction of an intermediary variable, the total effect of the independent variable on the dependent variable is weakened or even reversed. At this point, the intermediary variable does not transmit the influence of the independent variable on the outcome; instead, it suppresses or masks the effect of the direct effect, making it difficult to intuitively present the overall effect. On the contrary, the complementary effect is conducive to demonstrating the overall effect. As a result, in this study the total indirect effect is the sum of the complementary indirect effect and the competitive indirect effect. That is, 0.183+ (-0.023) = 0.160.

## Discussion

This study tested a chain mediation model to examine the mechanisms through which trait procrastination influences sleep quality among college students. The findings indicated that trait procrastination not only directly predicted poorer sleep quality but also exerted indirect effects through both independent and sequential mediation pathways involving self-efficacy and executive function. Specifically, higher levels of trait procrastination were associated with lower self-efficacy, impaired executive function, and worse sleep quality, suggesting that trait procrastination adversely affects not only mental health but also higher-order cognitive processes and physiological functioning.

### The relationship between trait procrastination and sleep quality

A significant positive correlation was observed between trait procrastination and poor sleep quality, supporting Hypothesis 1. The investigation focuses on the specific group of college students, who are faced with multiple challenges such as academic pressure and identity transformation. Within a short period, students must adapt to an autonomous university learning model. When students experience repeated academic setbacks, they may develop learned helplessness, leading to avoidance-based coping strategies. Over time, such maladaptive responses can become habitual, particularly in the absence of adequate social support. As a stable personality characteristic, trait procrastination exerts a sustained negative influence on individual’s mental and physical health. Students high in this trait often avoid demanding academic tasks and seek immediate gratification through activities such as smartphone use and short-form video consumption. Consequently, their impaired self-regulatory capacity disrupts circadian rhythms, leading to a habitual pattern of late-night sleep onset and a corresponding degradation in sleep quality.

### The mediating role of self-efficacy

Self-efficacy significantly mediated the relationship between trait procrastination and sleep quality, supporting Hypothesis 3. Although this pathway accounted for only 7.31% of the total effect, a masking (competitive) mediation was identified, suggesting that self-efficacy may suppress part of the negative impact of procrastination on sleep.

First, the significant negative correlation between trait procrastination and self-efficacy was well-established in the present study. This pattern is consistent with the concept of learned helplessness. When confronted with challenges, individuals with high trait procrastination typically adopt delaying and evasive strategies. This behavior deprives them of opportunities for a sense of accomplishment, thereby intensifying feelings of frustration and failure. Consequently, they begin to perceive tasks as excessively difficult and doubt their ability to succeed. This cycle gradually erodes their motivation to take initiative, fostering self-doubt and self-criticism, which in turn continuously undermines their self-efficacy.

Second, contrary to Hypothesis 2, self-efficacy was positively associated with poor sleep quality. A potential explanation for this counterintuitive finding lies in the distinct relationships that active and passive procrastination have with self-efficacy, which consequently lead to their differential impacts on sleep quality. Active procrastinators, who deliberately delay tasks as a strategic choice, typically exhibit normal or only slightly reduced self-efficacy and generally maintain adequate sleep quality. In contrast, passive procrastinators, who delay tasks due to an inability to act, often experience low self-efficacy. When they are faced with tasks that they cannot perform, only what they can do is leave it to next day. For them, bedtime may serve as an escape from tasks they feel incapable of completing, thereby paradoxically linking lower self-efficacy to better sleep quality.

### The mediating role of executive function

Executive function emerged as a key mediator in the relationship between trait procrastination and sleep quality, supporting Hypothesis 5. This pathway accounted for 55.27% of the total effect, indicating that executive dysfunction is a central mechanism through which procrastination impairs sleep.

First, the results indicate that not only was trait procrastination significantly negatively correlated with executive function, but age also emerged as a negative predictor (β = –0.070, p < 0.05), suggesting a gradual decline in these cognitive abilities among college students over time. These relationships can be interpreted through the flexibility theory of executive functions, which emphasizes their plasticity (86). This perspective is particularly relevant for college students, whose executive functions are in a stage of ongoing maturation and may be especially susceptible to adverse influences. When these developing cognitive systems are shaped by procrastinatory traits, individuals may enter a state of psychological and behavioral ambivalence, ultimately leading to diminished efficiency and collaborative capacity in executive processes. Our findings thus reinforce the critical and detrimental impact of procrastination on executive functioning during this formative period.

Second, executive function also significantly negatively predicted poor sleep quality, supporting Hypothesis 4. The result is consistent with the interplay between “hot” and “cool” executive functions. According to the dual-systems model, problematic behaviors stemming from impaired executive function arise from a dysregulation between the impulsive, emotional system and the analytical, cognitive system (103, 104). This creates a cognitive-affective dilemma where individuals are dominated by the “hot” system, pursuing immediate gratification while failing to engage the “cool” system required for self-regulation and long-term planning. From this perspective, sleep problems can be viewed as a manifestation of this self-regulatory failure. When confronted with the decision to end their day (“time-to-end threats”), individuals with deficient “cool” cognitive resources struggle to override the allure of social media and other engaging stimuli. Consequently, they succumb to distractions and fail to prioritize the biologically essential task of sleep.

### The chain mediating role of self-efficacy and executive function

This study also identified a significant, albeit small, chain mediation effect of self-efficacy and executive function in the relationship between trait procrastination and sleep quality, supporting H6. Despite its modest size, this effect may provide a valuable insight into the complex mechanisms at play.

First, the TDM theory of procrastination supports this finding, suggesting that such individuals are easily ensnared in a time management dilemma. Sleep, possessing the dual properties of time consumption and functional recovery, becomes a focal point for this conflict. Upon encountering “time-to-end threats,” a sense of urgency traps them (105). However, they remain acutely aware that time is passing and that sleep loss is non-productive, yet they cannot resolve the tension. This leads to engagement in avoidant, time-filling activities or a state of restless ambivalence in bed, caught between the need for sleep and task-related anxiety until exhaustion prevails. This nightly pattern of unresolvable conflict and failed disengagement solidifies into a habitual pre-sleep state. It is this self-perpetuating cycle of cognitive-emotional arousal and non-restorative coping that directly underpins the gradual deterioration of sleep quality.

Second, the chain mediation effect (trait procrastination → self-efficacy → executive function → sleep quality) is much weaker than the direct path (trait procrastination → executive function → sleep quality). This discrepancy suggests that self-efficacy may act not merely as a sequential mediator, but as a concurrent buffer. This “buffering hypothesis” is well-supported by extant literature. Self-efficacy, defined as confidence in one’s task-completion abilities, has been shown to mitigate the impact of procrastination. For instance, higher academic self-efficacy reduces the negative effect of academic procrastination on performance (106, 107). and it has been identified as the most potent mediator in reducing procrastination (108). Crucially, evidence confirms that self-efficacy beliefs can buffer the detrimental effects of low educational attainment on executive functioning (109). Consequently, we propose that self-efficacy does not only transmit the effect of procrastination but also directly buffers its impact on executive function and sleep quality, thereby explaining the smaller indirect effect through the mediated chain.

Third, older students reported better sleep quality than their younger counterparts when controlling for procrastination, self-efficacy, executive function, and gender. One potential explanation for this finding may lie in the distinction between active and passive procrastination. Older students may be more likely to engage in active procrastination—a strategic and deliberate delay of tasks which allows them to preserve sleep hygiene despite pending responsibilities. Alternatively, if they exhibit traits of passive procrastination, they may have developed more effective coping mechanisms over time, such as non-confrontational mentally disengaging from unfinished tasks, or an acceptance-oriented approach where they consciously defer tasks to the next day without undue rumination. As a result, their pre-sleep cognitive arousal was reduced. In either case, older students appear better equipped to mitigate the sleep-impairing effects typically associated with procrastination, thereby experiencing comparatively better sleep quality.

### Theoretical implications

First, as scholars have noted, few studies have explored the relationship between trait procrastination and sleep quality. Departing from prior research that primarily focused on risk factors, this study introduces a novel explanatory framework by investigating the mediating roles of positive individual traits and advanced cognitive functions. Individuals with high trait procrastination may tend to perceive sleep as both an interruption to work and a significant time cost (that means deadline is coming), thereby extending procrastinatory behaviors pattern into the domain of sleep regulation. These findings broaden the explanatory power of the TDM theory of procrastination (110), and might provide novel insights into the mechanisms through which trait procrastination influences sleep quality among college students.

Second, this research further identifies the key pathway through which trait procrastination influences sleep quality, specifically through the mediating role of executive function. This suggests that impaired executive function is a proximal factor predicting individuals’ poor sleep quality. This discovery further confirms the universal applicability of the Synergistic and Differentiation Theory of Executive Function. More importantly, this study has enriched the theoretical foundation of the field of trait procrastinators’ sleep quality among college students, and has provided potential new perspectives and directions for future research on related mechanisms and theoretical models.

Third, the mask effect of self-efficacy on the link between trait procrastination and sleep quality was observed in this investigation. This indicated that, when it comes to dealing with sleep problems, the self-efficacy of procrastinators may play a complex role. This result supplemented and enriched the procrastination–health model view. It might imply that procrastinators have stopped pushing themselves to complete the tasks that they cannot finish and accept the reality of sleep time lapse. This may be a self-protection mechanism with a potential attempt to break the cycle of procrastination and poor sleep quality. Therefore, future efforts should focus not only on executive function and integrated psychological functions, but also on addressing the self-efficacy of procrastinators in terms of sleep quality and achieving the optimal level.

### Practical implications

On the one hand, this research underscores the importance of executive function-based mental health programs in helping college students defend against the trait procrastination and poor sleep quality. Based on this, we recommend that procrastinators prioritize fostering executive function through innovative methods. For instance, a study provide evidence for the “fresh start effect” by showing that a temporal landmark signaling a new beginning helps speed up their task completion. Notably, we demonstrate that the “fresh start nudge” can facilitate early task completion through the underlying processes of meaningfulness and motivation (111).

On the other hand, maintaining an optimal and balanced level of self-efficacy is important for procrastinators. It is true that a high level of self-efficacy is good for the task to be carried out and completed, and self-efficacy for sleep hygiene is a modifiable factor that may serve to improve sleep quality (112). Hower, unrealistic self-efficacy will not help things go smoothly, including sleep. The current study revealed a double-edged effect of self-efficacy on procrastination (113). In this paper, we argue that it is particularly important to recognize the waxing and waning of intentions. Intentions may sometimes be temporarily put aside rather than being truly abandoned (114). Therefore, it is necessary to help procrastinators recognize their true level of ability to find the most suitable way.

## Limitations and perspectives

This study is subject to several limitations that should be considered when interpreting the results.

First, the use of convenience sampling (e.g., university students, online panels) fundamentally challenges the external validity of the findings, primarily due to the lack of randomness. This approach introduces significant biases, including selection bias, sample homogeneity, and volunteer bias, thereby limiting the generalizability of the results to broader populations. Future research should employ rigorous random sampling techniques and include participants from diverse age groups and cultural backgrounds to examine the cross-group and cross-cultural applicability of the proposed framework. For example, students with a special extra-academic activity such as working or playing music could determine different level of self-efficacy and consequent sleep quality (115).

Second, although the online survey methodology facilitated efficient data collection, it may have introduced bias related to digital literacy and internet access. Individuals with limited technological proficiency, inadequate internet connectivity, or from specific demographic groups (e.g., older adults or certain socioeconomic backgrounds) are likely to be underrepresented. This may affect the representativeness of the sample and the generalizability of the findings. Future studies could adopt mixed-methods approaches to capture perspectives that may be marginalized in purely digital formats.

Third, all measures in this study were based on self-reported data, which are susceptible to social desirability bias. The incorporation of objective assessment methods in future research would strengthen the validity of the findings.

Fourth, the cross-sectional design precludes causal inferences regarding the relationship between trait procrastination and sleep quality. Experimental interventions and longitudinal follow-ups are recommended to elucidate the long-term and causal effects.

Finally, this study did not fully account for potential confounding variables, such as academic stress, socioeconomic status, social support, or comorbid mental disorders (e.g., depression and anxiety), that may influence sleep quality. The absence of controls for these factors may compromise the validity and interpretation of the results. Additionally, the lack of detailed demographic information (e.g., academic major and family background) further limits the representativeness of the sample. Future studies should systematically collect and control for these variables to enhance the robustness and generalizability of the findings.

## Conclusion

The main findings of this study are: 1) Trait procrastination significantly negatively predicts sleep quality. 2) Self-efficacy mediates the relationship between trait procrastination and sleep quality. 3) Executive function mediates the relationship between trait procrastination and sleep quality. 4) Self-efficacy and executive function played a chain mediation role between trait procrastination and sleep quality. 5) Self-efficacy plays a masking effect on the relationship between trait procrastination and sleep quality. That means self-efficacy does not convey the influence of trait procrastination on sleep quality. Instead, it suppresses or masks the direct relationship between trait procrastination and sleep quality. Interventions aimed at improving executive function have great potential to enhance the sleep quality of college students with procrastination problems. And maintaining an optimal and balanced level of self-efficacy is important for these procrastinators to improve sleep quality.

## Data Availability

The raw data supporting the conclusions of this article will be made available by the authors, without undue reservation.
